# The Environmental Pollutant Cadmium Promotes Influenza Virus Replication in MDCK Cells by Altering Their Redox State

**DOI:** 10.3390/ijms14024148

**Published:** 2013-02-19

**Authors:** Paola Checconi, Rossella Sgarbanti, Ignacio Celestino, Dolores Limongi, Donatella Amatore, Alessandra Iuvara, Alessandro Alimonti, Enrico Garaci, Anna Teresa Palamara, Lucia Nencioni

**Affiliations:** 1 Department of Public Health and Infectious Diseases, Institute Pasteur, Cenci-Bolognetti Foundation, “Sapienza” University of Rome, Rome 00185, Italy; E-Mails: paola.checconi@uniroma1.it (P.C.); ignacio.celestino@uniroma1.it (I.C.); donatella.amatore@uniroma1.it (D.A.); lucia.nencioni@uniroma1.it (L.N.); 2 San Raffaele Pisana Scientific Institute for Research, Hospitalization and Health Care, Rome 00163, Italy; E-Mail: rossella.sgarbanti@uniroma1.it; 3 Department of Experimental Medicine and Surgery, University of Rome “Tor Vergata”, Rome 00133, Italy; E-Mails: doloreslimongi@gmail.com (D.L.); alessandra.iuvara@ptvonline.it (A.I.); garaci@uniroma2.it (E.G.); 4 Department of Environment and Primary Prevention, Italian National Institute for Health, Rome 00161, Italy; E-Mail: alessandro.alimonti@iss.it

**Keywords:** cadmium, pollutants, influenza virus infection, oxidative stress, glutathione, respiratory diseases

## Abstract

Cadmium (Cd) is a toxic heavy metal that is considered an environmental contaminant. Several sources of human exposure to Cd, including employment in primary metal industries, production of certain batteries, foods, soil and cigarette smoke, are known. Its inhalation has been related to different respiratory diseases and toxic effects, among which alterations of the physiological redox state in individuals exposed to the metal have been described. Host-cell redox changes characteristic of oxidative stress facilitate the progression of viral infection through different mechanisms. In this paper, we have demonstrated that pre-treatment with CdCl_2_ of MDCK cells increased influenza virus replication in a dose-dependent manner. This phenomenon was related to increased viral protein expression (about 40% compared with untreated cells). The concentration of CdCl_2_, able to raise the virus titer, also induced oxidative stress. The addition of two antioxidants, a glutathione (GSH) derivative or the GSH precursor, *N*-acetyl-l-cysteine, to Cd pre-treated and infected cells restored the intracellular redox state and significantly inhibited viral replication. In conclusion, our data demonstrate that Cd-induced oxidative stress directly increases the ability of influenza virus to replicate in the host-cell, thus suggesting that exposure to heavy metals, such as this, could be a risk factor for individuals exposed to a greater extent to the contaminant, resulting in increased severity of virus-induced respiratory diseases.

## 1. Introduction

Cadmium (Cd) is a toxic heavy metal and, as a byproduct of smelters, is a prevalent environmental contaminant. There are several sources of human exposure to Cd, including employment in primary metal industries, production of certain batteries, in foods and soil [1]. It is also present in cigarette smoke, and its inhalation is associated with decreased pulmonary function, lung cancer and chronic obstructive pulmonary disease [2]. Cadmium accumulation in many organs, such as kidney, liver, lung, testis, bone and the blood system, is extremely toxic [3]. The molecular mechanisms, which cause the toxic effects of Cd, involve interference with antioxidant enzymes, alteration in thiol proteins, inhibition of energy metabolism, alteration in DNA structure and modification of some enzyme activities [3]. In particular, it induces depletion of reduced glutathione (GSH) and protein-bound sulfhydryl groups, resulting in enhanced production of reactive oxygen species (ROS) [4–6]. Physiologically, antioxidant defense systems of the cell minimize the changes caused by ROS, which play a role in the regulation of many intracellular signaling pathways as activators of signal transduction [7]. When ROS generation is increased to an extent that exceeds the antioxidant activity of specific enzymes and molecules, it results in oxidative stress [8], causing damage to DNA, proteins or mitochondria, lipid peroxidation and apoptotic cell death [9].

Numerous studies have reported that viral infection is often associated with redox changes characteristic of oxidative stress. Indeed, a shift towards an oxidized state has been observed in the cells and body fluids of patients infected with human immunodeficiency virus (HIV) and the hepatitis C virus (reviewed in [10]). Moreover, an alteration of the endogenous levels of GSH has been observed *in vitro* in experimental infections with herpes simplex virus type 1 (HSV-1) [11], parainfluenza virus [12] and HIV [13], as well as *in vivo* in HSV-1 and HIV infection [14,15]. A decrease in GSH levels and general oxidative stress has also been demonstrated during influenza virus infection in both *in vitro* and *in vivo* experimental models [16–20]. In particular, the bronchoalveolar lavage fluid from infected mice showed increased production of superoxide [21], increased activity of the superoxide-generating xanthine oxidase enzyme [22], decreased concentrations of GSH and increased levels of oxidized glutathione (GSSG) and malondialdehyde, which is an indicator of lipid peroxidation [23].

Several compounds able to induce oxidative stress have been demonstrated to favor susceptibility to viral infections [20,24,25]. In particular, cocaine [26], as well as morphine [27], increased parainfluenza virus replication by depletion of the intracellular GSH of infected cells. Jaspers *et al.* [28] reported that in human respiratory epithelial cells, oxidative stress generated by diesel exhaust (DE) increased the susceptibility to influenza infection and that exposure to DE increased the ability of the virus to attach and enter respiratory epithelial cells. The addition of the antioxidant GSH-ethyl ester increased cellular GSH levels and reversed the effects of DE on influenza virus infection.

On the basis of this evidence and on the fact that not only exposed workers, but also environmentally exposed populations that are at risk of severe health problems, including pulmonary diseases, we hypothesized that exposure to Cd may directly contribute to enhance influenza virus replication by altering the redox balance of infected cells. In this paper, we have demonstrated that pre-treatment with CdCl_2_ of Madin Darby Canine Kidney (MDCK) cells induced: (i) an imbalance in the redox state *versus* an oxidized state; (ii) an increase in viral protein synthesis and, as a consequence, an increase in virus release from infected cells; and (iii) the addition of two antioxidants, a GSH derivative (GSH-C4) or the GSH precursor, *N*-acetyl-l-cysteine (NAC), to Cd-treated and infected cells significantly inhibited viral replication.

## 2. Results

### 2.1. CdCl_2_ Was Not Toxic for Cells until the Concentration of 50 μM

**Figure 1:**
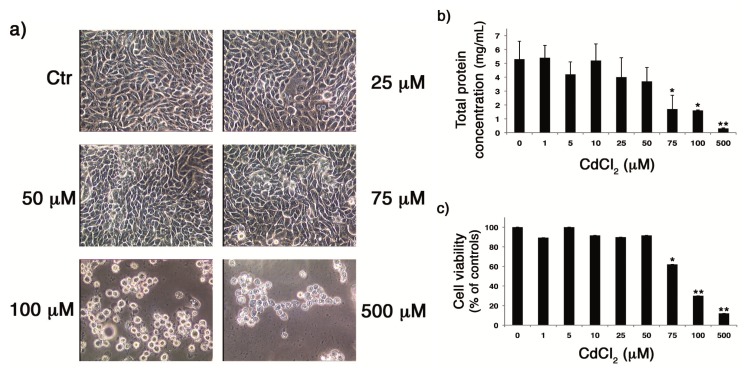
Toxic effects of CdCl_2_ on Madin Darby Canine Kidney (MDCK) cells. (**a**) Microscopic examination of MDCK cell monolayers treated with different concentrations of CdCl_2_ (0, 25, 50, 75, 100 and 500 μM) for 18 h. (**b**) Total protein concentration measured using the Bradford assay in lysates of MDCK cells after treatment with different concentrations of CdCl_2_ (0, 1, 5, 10, 50, 75, 100 and 500 μM) for 18 h. Each value is the mean ± SD of four experiments, each run in duplicate. (**c**) MTT assay of MDCK cells after treatment with different concentrations of CdCl_2_ (0, 1, 5, 10, 50, 75, 100 and 500 μM) for 18 h. The values of cell viability are expressed as percentages of Cd-treated *versus* untreated cells. Each value is the mean ± SD of two experiments, each run in triplicate. ^*^p < 0.005, ^**^p < 0.001 *versus* untreated cells.

In the first set of experiments, the eventual cytotoxicity of CdCl_2_ on MDCK cells by phase contrast microscope analysis was evaluated. Confluent monolayers were treated with different CdCl_2_ concentrations (range 25–500 μM) and incubated for 18 h. As shown in Figure 1a, no evident alterations in the monolayer were found when the compound was added at concentrations of 25 and 50 μM, compared with untreated cells. By contrast, the administration of high doses of CdCl_2_ caused evident signs of cytotoxicity. Cells treated with 75 μM CdCl_2_ appeared rounded and lost intercellular contact, and most of the cells were detached at concentrations of 100 and 500 μM, indicating a high rate of cell death.

In order to assess whether the toxic effect of CdCl_2_ was associated with altered protein synthesis, total protein concentration obtained from lysates of cells treated with different concentrations of CdCl_2_ was measured by means of the Bradford assay. As shown in Figure 1b, treatment with low concentrations of CdCl_2_ (1–50 μM) did not cause significant effects on cellular protein synthesis compared to untreated cells, while a reduction in protein synthesis was measured at high concentrations. In particular, the addition of CdCl_2_ at concentrations of 75, 100 and 500 μM significantly decreased protein concentrations (63.5% ± 6%, 72.5% ± 4% and 94.6% ± 0.4%, respectively) in comparison to untreated cells. Diminished protein synthesis may be considered as an index of reduced cell number, thus these results suggest that, at high doses, CdCl_2_ causes cell death.

Finally, the effect of CdCl_2_ on the metabolic activity of the cells was evaluated using the MTT assay. As shown in Figure 1c, treatment with 1–50 μM CdCl_2_ did not cause alterations on cell viability, while higher doses (75–500 μM) of CdCl_2_ significantly reduced proliferation of the cells with respect to untreated cells. Thus, due to the toxicity of CdCl_2_ at high doses, we performed the following experiments within a range from 1 to 50 μM.

### 2.2. CdCl_2_ Alters the Intracellular Redox Balance *versus* an Oxidized State

**Figure 2:**
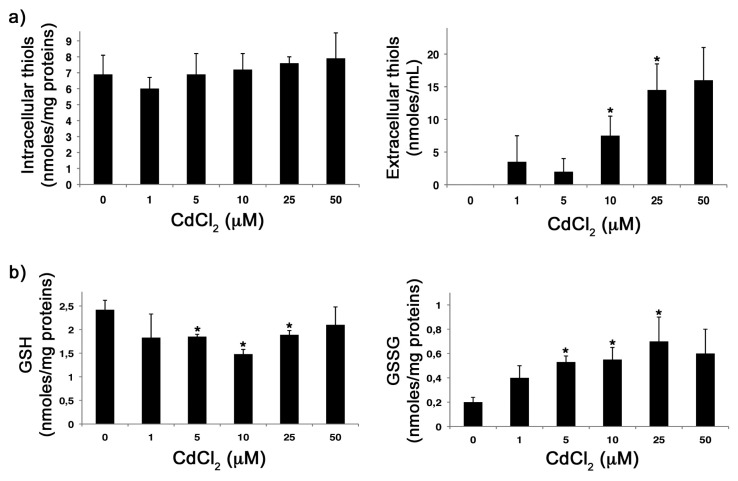
Effect of CdCl_2_ on the intra- and extra-cellular redox balance. (**a**) Intra- and extra-cellular thiol levels (left and right graphs, respectively) were measured in MDCK cells treated for 18 h with different concentrations of CdCl_2_ (0, 1, 5, 10, 25 and 50 μM), as described in the Methods section. (**b**) Intracellular reduced glutathione (GSH) and oxidized glutathione (GSSG) content (left and right graphs, respectively) were measured in MDCK cells treated for 18 h with different concentrations of CdCl_2_ (0, 1, 5, 10, 25 and 50 μM), as described in the Methods Section. Each value is the mean ± SD of two separate experiments, each performed in duplicate. ^*^p < 0.01 *versus* untreated cells.

It is known that the molecular mechanisms underlying the toxic effects of Cd include interference with antioxidant enzymes, thiol protein modification and GSH depletion [3]. To evaluate whether Cd was able to alter the intracellular redox state in our experimental system, confluent monolayers of MDCK cells were treated with the CdCl_2_ at doses that were not toxic and incubated for 18 h. Then, levels of free thiols, as well as GSH and GSSG content, were measured. As shown in Figure 2a (left), with respect to untreated cells, no significant differences were observed in the levels of intracellular thiols in cells treated with CdCl_2_, while an increase in extracellular thiols released in the supernatant of the same cells was measured (Figure 2a, right). Furthermore, treatment with the metal caused a decrease in intracellular GSH levels (Figure 2b, left), as well as an increase in GSSG content (Figure 2b, right). These results indicate that CdCl_2_ is able to alter the redox balance *versus* an oxidized state, especially by altering the GSH/GSSG ratio.

### 2.3. CdCl_2_ Induces an Increase in Influenza Virus Replication

**Figure 3:**
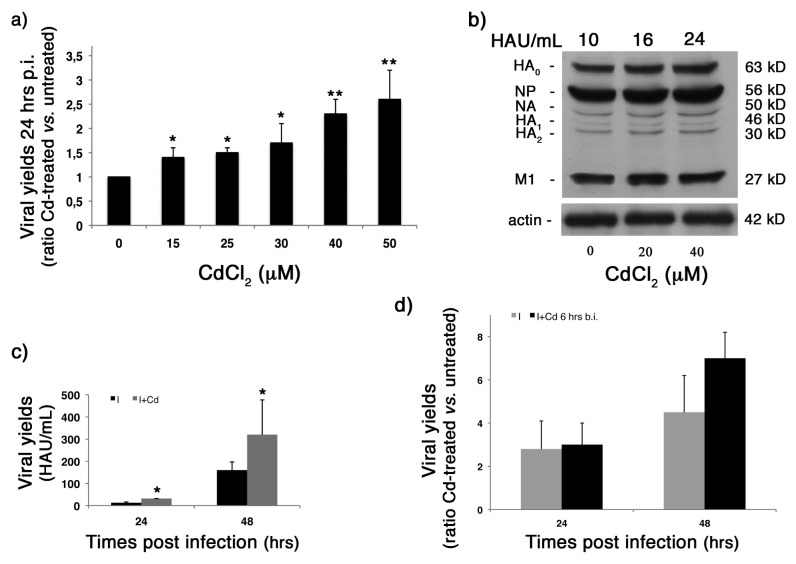
Effect of CdCl_2_ on influenza virus replication. (**a**) Viral yields in MDCK cells treated with different concentrations of CdCl_2_ (0, 15, 25, 30, 40 and 50 μM) 18 h before infection (b.i.) with human influenza virus A/Puerto Rico/8/34 H1N1 (PR8) (0.5 multiplicity of infection (m.o.i.)). The 24 h post-infection (p.i.) viral yields are expressed as the ratio between hemagglutinin units (HAU) values obtained from Cd-treated cells *versus* those recorded for control cells. Each value is the mean ± SD of four experiments, each run in duplicate. (**b**) Western blot analysis in lysates of MDCK cells treated with CdCl_2_ (0, 20 and 40 μM) 18 h b.i. Proteins were separated by 12% sodium dodecyl sulfate polyacrylamide gel electrophoresis (SDS-PAGE), transferred onto nitrocellulose membrane and immunostained with anti-influenza antibody (Ab). PR8 virus proteins and molecular weight values are indicated to the left and to the right of the figure, respectively. The same nitrocellulose filter was stripped and re-stained with anti-actin Abs. Viral yields, expressed as hemagglutinin units (HAU)/mL are reported in the upper panel of figure. (**c**) Viral yields in MDCK cells treated with CdCl_2_ (0 and 40 μM) 18 h b.i. The 24 and 48 h p.i. viral yields are expressed as hemagglutinin units (HAU)/mL. Each value is the mean ± SD of two experiments, each run in duplicate. (**d**) Viral yields in MDCK cells treated with CdCl_2_ (0 and 50 μM) 6 h b.i. The 24 and 48 h p.i. viral yields are expressed as the ratio between HAU values obtained from Cd-treated cells *versus* those recorded for control cells (I: Infected). Each value is the mean ± SD of two experiments, each run in duplicate. ^*^p < 0.05, ^**^p < 0.005 *versus* untreated cells.

Several molecules able to induce oxidative stress have been demonstrated to increase viral replication [26,27,29], including influenza virus [18,20]. Thus, to verify whether the CdCl_2_-induced redox imbalance was able to promote influenza virus replication, confluent monolayers of MDCK cells were pre-treated with CdCl_2_ at different concentrations for 18 h, then cells were infected with human influenza virus A/Puerto Rico/8/34 H1N1 (PR8 virus) for 24 h (as described in the Methods section), and viral replication was evaluated in cell supernatants by hemagglutination assay. As shown in Figure 3a, pre-treatment with CdCl_2_ significantly increased viral production in a dose-dependent manner (range of increase: 1.4–2.6-times in Cd-treated cells, compared with untreated cells). Then, to evaluate whether the increase in viral replication observed in Cd-treated cells was related to an increase in viral protein synthesis, two doses (20 and 40 μM) of CdCl_2_ were used, as representative of the lower and higher concentrations of the metal. As shown in Figure 3, in Cd-treated cells, the increase in virus yields (upper panel, Figure 3b) was related to an increase in viral protein synthesis (Figure 3b). In particular, densitometric analysis of viral protein expression, normalized for actin, revealed an increase of protein synthesis of 24% ± 7% and 37% ± 14% in cells treated with 20 and 40 μM CdCl_2_, respectively, compared with untreated cells.

To evaluate whether this phenomenon was due to an enhanced production of viral particles in a given time or to an accelerated virus release, a kinetic of viral replication was performed. For this purpose, a virus-induced cytopathic effect was observed until 48 h post-infection (p.i.), and the supernatant of infected cells was recovered at 24 and 48 h p.i., both in untreated and Cd-treated cells. As shown in Figure 3c, in Cd-treated cells, the increase in viral production was stable as long as 48 h p.i., thus suggesting that it was not related to an accelerated virus release. Moreover, Cd had no effect on influenza virus replication when added immediately after infection and incubated for the following 24 h or when it was added 30 min, 1–2 and 4 h before infection (b.i.) (data not shown). A slight, but no significant, increase was observed only when cells were treated with the highest concentration of Cd 6 h b.i. (Figure 3d), thus suggesting that an incubation time of the metal is needed to make cells more permissive to viral replication.

Our results indicate that the addition of CdCl_2_ induces a more favorable environment for viral replication into the cells. In other words, it increases their permissiveness to viral infection.

### 2.4. Treatment with Antioxidants Reverts the CdCl_2_-Induced Increase of Influenza Virus Replication

**Figure 4:**
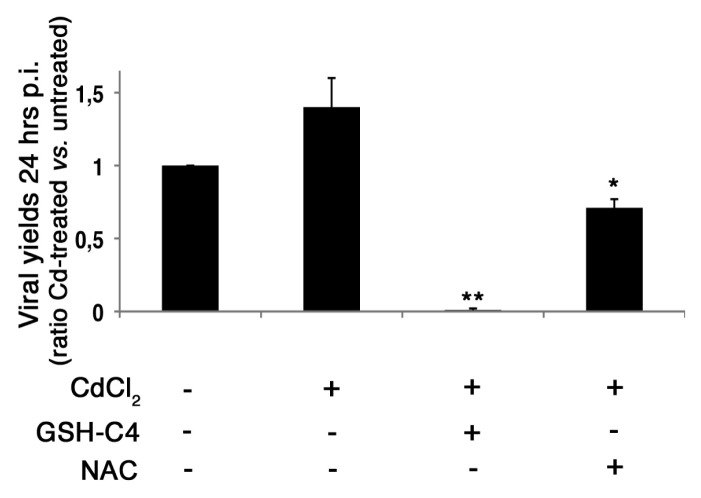
Effect of antioxidants on influenza virus replication. Viral yields in MDCK cells treated with 25 μM CdCl_2_ 18 h before PR8 infection (0.5 m.o.i.) and treated with butanoyl GSH-derivative (GSH-C4) (10 mM) or *N*-acetyl-l-cysteine (NAC) (5 mM). The 24 h p.i. viral yields are expressed as the ratio between HAU values obtained from Cd-treated cells *versus* those recorded for infected (control) cells. Each value is the mean ± SD of two experiments, each run in duplicate. ^*^p < 0.05, ^**^p < 0.01 *versus* Cd-treated cells.

To determine whether Cd-induced increase in cell permissiveness to influenza virus was linked to the modulation of the intracellular redox state, confluent monolayers of MDCK cells were pre-treated with CdCl_2_ for 18 h. Cells were then infected with PR8 and treated with two antioxidants: the, butanoyl GSH-derivative (GSH-C4) or the GSH precursor, *N*-acetyl-l-cysteine (NAC). After 24 h, as expected, when cells were pre-treated with CdCl_2_, viral production increased compared to infected (untreated) cells, considered as control cells (Figure 4). On the contrary, treatment with the antioxidants to Cd-treated cells hampered the metal-induced increase in viral replication (about 99.3% and 49.3% reduction in GSH-C4-treated or in NAC-treated cells, respectively, compared with cells treated only with the metal). Interestingly, the addition of GSH-C4 was able to reduce the viral titer (about 99% inhibition) compared with infected (control) cells, thus suggesting a multiple action of this antioxidant in regulating viral replication.

Overall our data indicate that CdCl_2_-induced redox changes in the cells are able to directly promote viral replication, probably by activating various redox-regulated pathways that are important for the virus life cycle [20].

### 2.5. Discussion

In this paper, we have reported that exposure to the heavy metal, Cd, makes epithelial cells more permissive to influenza A virus infection. In detail, we have demonstrated that: (i) the treatment of MDCK cells with Cd altered the intracellular redox balance *versus* an oxidized state; (ii) in parallel with the Cd-induced redox imbalance, viral replication increased with respect to that found in control conditions, and this effect was maintained until 48 h p.i.; and (iii) recovery of reduced conditions in Cd-treated cells with different antioxidants led to a significant inhibition of viral replication. In particular, this reduction was more evident in cells treated with GSH-C4.

The toxic mechanisms of Cd are not well understood, but it is known that they act mainly via free radical-induced damage, particularly in the lungs, kidneys, bone, central nervous system, reproductive organs and heart [30]. Indeed, increased production of ROS, depletion of GSH and protein-bound sulfhydryl groups have been reported in different models, both *in vivo* and *in vitro* [31]. With regard to GSH, Cd shows a high affinity for this antioxidant [32], which is a primary intracellular antioxidant and conjugating agent and accounts for up to 90% of the total low molecular weight cellular thiols [33]. GSH acts by scavenging Cd to prevent its interaction with specific cellular targets and, in our model, treatment with Cd for 18 h induced GSH depletion, thus suggesting a direct interaction between the metal and intracellular GSH. However, we cannot exclude the possibility that exposure to Cd may lead to changes in the activity of enzymes involved in GSH biosynthesis. Indeed, an inhibition of glutathione peroxidase, transferase and reductase activity has been reported *in vitro* and *in vivo* experimental models after Cd exposure [34–36]. On the other hand, in Cd-treated cells, GSH content, its homeostasis and synthesis, may depend on the timing of exposition (acute or chronic) and the dose of Cd [32,37–39]. Indeed, some authors have reported that, after exposure to higher concentrations of CdCl_2_, GSH levels increased with respect to that observed after exposure to lower doses, suggesting a protective role of this cellular antioxidant in response to the oxidative stress induced by the treatment [37]. In our model, cells treated with high doses of Cd (see Figure 2b) showed a slight recovery in GSH content, which corresponded to a reduction of GSSG. We can hypothesize that, at high doses, cells may counteract depletion of GSH by increasing GSH biosynthesis. Further studies will be necessary to deepen this trend.

Interestingly, oxidative stress influences the functionality of several signaling pathways that are hijacked by viruses to ensure their own replication and/or regulate inflammatory responses and the fate of infected cells [29]. Virally-induced oxidative stress seems to exert direct effects on influenza virus replication, by activating redox-regulated p38 MAPK (Mitogen Activated Protein Kinase) activation [40]. This latter facilitates nuclear export of the viral ribonucleoprotein (vRNP) complexes, through phosphorylation of the viral nucleoprotein (NP), an event that is essential for the protein’s translocation from the nucleus to the cytoplasm [41]. The nuclear-cytoplasmic traffic of vRNP is blocked by the natural phenol, resveratrol, or its synthetic analogues, through inhibition of the protein kinase C (PKC) phosphorylation and its dependent pathways, c-Jun NH_2_-terminal kinase (JNK) and p38 MAPK, leading to a significant reduction in influenza virus replication [42,43]. Cadmium is known to activate PKC, which results in enhanced phosphorylation of various transcription factors and activation of target gene expression [30]. Moreover, it has been reported to activate p38 MAPK, JNK and extracellular signal-regulated kinases (ERK) in different cell types [44–48]. We found that Cd was able to increase viral protein synthesis and virus titer released from infected cells. The mechanisms involved are still unknown, however it is reasonable to hypothesize that, in our model, the redox imbalance induced by Cd might contribute to the activation of some redox-sensitive cascades that regulate viral replication. It is also possible that the treatment with Cd might upregulate pathways involved in viral mRNA transcription or in the acceleration of translation. All these events make the cells more permissive to influenza virus. In order to fully understand the molecular mechanisms underlying the effects of Cd on viral replication, further studies will be necessary.

The addition of antioxidants, such as GSH, its derivatives or precursors, is able to inhibit replication of several DNA or RNA viruses [11,12,49,50]. With regard to influenza virus, we demonstrated that GSH-C4 strongly inhibited influenza A virus replication in cultured cells and in lethally infected mice [20]. In particular, we demonstrated that maturation of viral hemagglutinin (HA) glycoprotein was dependent on a host-cell oxidoreductase, the protein disulfide isomerase (PDI), whose activity in infected cells was probably facilitated by virus-induced GSH depletion. By correcting this deficit, GSH-C4 increased levels of reduced PDI and inhibited essential disulfide bond formation in HA, leading to an impairment of viral particle maturation [20]. In the present paper, the addition of GSH-C4 or NAC hampered the increase of viral yields observed during Cd treatment (see Figure 4). Interestingly, GSH-C4 significantly reduced viral replication compared with (infected) control cells. These results strongly suggest the hypothesis that this antioxidant could exert a dual action: (a) it restores reduced conditions in cells treated with Cd, thus making cells more resistant to viral infection; and (b) it prevents GSH depletion, typically induced by viral infections, thus impairing viral replication.

Overall, the data confirm the relevance of the intracellular redox balance in regulating viral replication, and exposure to substances able to induce oxidative stress may contribute to increasing susceptibility to viral infections. This argument is particularly relevant for respiratory viruses that are a major cause of pulmonary-related illnesses in children, the elderly and susceptible populations, such as individuals with chronic obstructive pulmonary diseases and immunocompromised patients. Moreover, several studies have suggested that the level of air pollution that is common in many urban and industrial environments is an important risk factor for various adverse health effects in humans, including respiratory diseases [51]. Among air pollutants, DE has been shown to reduce host defenses, resulting in decreased resistance to respiratory infections. In particular, a recent study has shown that exposure to DE during an influenza virus infection in mice polarized the local immune responses to an IL-4 dominated profile in association with increased viral disease, and the treatment with NAC blocked the DE-induced changes in cytokine profiles and lung inflammation [52]. It is worthwhile highlighting that emissions from diesel engines were claimed to be responsible for the substantial amounts of metals (e.g., 50% of quantified Cd) in ultrafine particles (<100 nm) observed along a roadside with heavy traffic [53].

## 3. Experimental Section

### 3.1. Cell Cultures

Madin-Darby Canine Kidney (MDCK) epithelial cells are a cell line permissive to influenza virus replication [54,55] and have been characterized in the past for their intracellular GSH content during viral infection [18,26,27]. Cells were grown in Roswell Park Memorial Institute (RPMI)-1640 medium, supplemented with 10% fetal bovine serum (FBS), glutamine 0.3 mg/mL, penicillin 100 U/mL and streptomycin 100 mg/mL. Cell viability was estimated by trypan blue (0.02%) exclusion. All reagents were purchased from Invitrogen (Milan, Italy).

### 3.2. Cytotoxicity Assay

Cytotoxicity of cadmium chloride (CdCl_2_ anhydrous, MW 183.3, purchased from Sigma-Aldrich, St. Louis, MO, USA) was evaluated by means of two different assays, one that measures total protein concentration and one that measures the cell metabolic activity. CdCl_2_ was dissolved in water and diluted to final concentrations in RPMI medium before use.

The Bio-Rad protein assay is a dye-binding assay in which a differential color change of a dye occurs in response to various concentrations of protein [56]. The absorbance maximum for an acidic solution of Coomassie Brilliant Blue dye shifts from 465 to 595 nm, when binding to basic and aromatic amino acid residues occurs. Briefly, cells were plated in a 60 mm tissue culture dish at 1 × 10^5^/mL for 24 h, and then, CdCl_2_ was added at different concentrations, ranging from 1 to 500 μM. After 18 h, cells were washed with cold PBS, centrifuged at 700× *g* for 10 min, and the pellet was lysed in 100 μL cold lysis buffer (10 mM Tris, 150 mM NaCl and 0.25% NP-40, pH 7.4) containing protease and phosphatase inhibitor mixtures. An equal amount of lysate was used for protein assay, and absorbance was measured at 595 nm. A standard curve was obtained with different concentrations of bovine serum albumin.

The 3-[4,5-dimethylthiazol-2-yl]-2,5-diphenyl-2*H*-tetrazoliumbromide (MTT, Sigma-Aldrich, St. Louis, MO, USA) is a pale yellow substrate that is cleaved by active mitochondria in living cells to yield a dark blue formazan product. Briefly, MDCK cells were plated in a 96 well-plate at a concentration of 2 × 10^4^/mL in RPMI-1640 without phenol red, supplemented with 10% FBS. After 24 h plating, CdCl_2_ was added at different concentrations (range 1–500 μM), and cells were incubated at 37 °C for 18 h. Cells were then incubated at 37 °C for 3 h with MTT (1 mg/mL). Following incubation, the remaining water insoluble formazan was solubilized in absolute isopropanol containing 0.1 N HCl. Absorbance of converted dye was measured in an ELISA plate reader at the wavelength of 570 nm. The cytotoxicity of the compounds was calculated as the percentage reduction of the viable cells compared with the drug-free control culture.

### 3.3. Virus Production, Infection and Titration

Human influenza virus A/Puerto Rico/8/34 H1N1 (PR8 virus) was grown in the allantoic cavities of 10-day-old embryonated chicken eggs. Cells were challenged 24 h after plating with PR8 at a multiplicity of infection (m.o.i.) of 0.5. Mock infection was performed with the same dilution of allantoic fluid from uninfected eggs. Infected and mock-infected cells were incubated for 1 h at 37 °C, washed with PBS and then incubated with medium, supplemented with 2% FBS. Virus production was determined in cell supernatants by measuring hemagglutinin units (HAU) at different times post-infection (p.i.), according to standard procedures.

### 3.4. Immunoblotting

Cells were washed with cold PBS, centrifuged at 700× *g* for 10 min, and the pellet was lysed in cold lysis buffer (10 mM Tris, 150 mM NaCl and 0.25% NP-40, pH 7.4) containing protease and phosphatase inhibitor mixtures. After 30 min on ice, lysates were centrifuged at 10,000× *g* for 30 min at 4 °C, and total protein concentration was determined using the Bradford protein assay (Bio-Rad, Milan, Italy). Cell lysates were re-suspended in sodium dodecyl sulfate (SDS) sample buffer containing 10% DL-dithiothreitol, separated with SDS-PAGE and blotted onto nitrocellulose membranes. The membranes were blocked with 10% nonfat dry milk in PBS-Tween for 1 h at room temperature. Goat polyclonal anti-influenza A virus Abs (Chemicon (DBA), Milan, Italy) were used as primary Abs. Secondary Abs were horseradish-peroxidase-conjugated (Amersham Biosciences (GE Healthcare), Milan, Italy). Blots were developed with an enhanced chemiluminescence system (Amersham Biosciences (GE Healthcare), Milan, Italy).

### 3.5. Quantitative Determination of Thiols

The total amount of intra- and extra-cellular free thiols of infected cells was determined by a standard colorimetric assay by using Ellman’s reagent (Sigma-Aldrich, St. Louis, MO, USA).

### 3.6. Glutathione Assay

Intracellular glutathione (GSH) and its oxidized form (GSSG) were measured using a glutathione assay kit (Sigma-Aldrich, St. Louis, MO, USA). The samples were first deproteinated with 5% 5-sulfosalicylic acid solution, then the GSH content of the samples was measured using a kinetic assay in which catalytic amounts of GSH cause a continuous reduction of 5,5′-dithiobis-(2-nitrobenzoic) (DTNB) acid to 5-thio-2-nitrobenzoic acid (TNB). The mixed disulfide, GSTNB (between GSH and TNB) that is concomitantly produced, is reduced by glutathione reductase to recycle the GSH and produce more TNB. The rate of TNB production, detected at 412 nm, is directly proportional to this recycling reaction that is, in turn, directly proportional to the concentration of GSH in the sample. For GSSG quantification, an aliquot of deproteinated samples was first incubated with 2-vinylpyridine at room temperature for 1 h to derivatize GSH. Values were expressed as nanomoles of GSH/GSSG per milligram of protein in the original cell extract.

### 3.7. Statistical Analyses

Unpaired data were analyzed using the Student *t* test and *p* values <0.05 were considered significant. Data are presented as the mean ± standard deviation (SD).

## 4. Conclusions

Our data indicate that exposure to substances able to induce oxidative stress favor viral replication. Thus, heavy metals, like Cd, could contribute to the development of respiratory diseases, and preventive strategies are needed, especially for people who are exposed to this contaminant to a greater extent.
